# Social Determinants of Health and Informed Consent Comprehension for Pediatric Cancer Clinical Trials

**DOI:** 10.1001/jamanetworkopen.2023.46858

**Published:** 2023-12-11

**Authors:** Paula Aristizabal, Shilpa Nataraj, Arissa K. Ma, Nikhil V. Kumar, Bianca P. Perdomo, Maria Elena Martinez, Jesse Nodora, Lin Liu, Euyhyun Lee, Courtney D. Thornburg

**Affiliations:** 1 Division of Pediatric Hematology and Oncology, Department of Pediatrics, University of California, San Diego, La Jolla; 2 Peckham Center for Cancer & Blood Disorders, Rady Children’s Hospital San Diego, San Diego, California; 3 Division of Population Sciences, Disparities and Community Engagement, University of California San Diego Moores Cancer Center, La Jolla, California; 4 Dissemination and Implementation Science Center, University of California, San Diego, Altman Clinical and Translational Research Institute, La Jolla; 5 School of Medicine, University of California, San Diego, La Jolla; 6 currently affiliated with Department of Pediatrics, Division of Hematology Oncology, Stanford University, Stanford, California; 7 currently affiliated with Department of Family Medicine, Kaiser Permanente, Long Beach, California; 8 currently affiliated with Department of Pediatrics, University of California Irvine, Irvine, California; 9 Herbert Wertheim School of Public Health, University of California, San Diego, La Jolla; 10 Altman Clinical and Translational Research Institute, University of California, San Diego, La Jolla

## Abstract

**Importance:**

Ensuring valid informed consent (IC) prior to enrollment in clinical trials is a fundamental ethical right.

**Objective:**

To assess whether social determinants of health (SDOH) and related sociocontextual factors are associated with parental IC comprehension in therapeutic childhood cancer clinical trials.

**Design, Setting, and Participants:**

This cross-sectional study prospectively enrolled 223 parents of children with newly diagnosed cancer at Rady Children’s Hospital San Diego, a large quaternary academic center in California, from October 1, 2014, to March 31, 2021. Linear mixed effects models were used to assess whether IC comprehension overall and by domain (purpose, procedures, and randomization; risks and benefits; alternatives; and voluntariness) were associated with SDOH and sociocontextual factors. Data were analyzed from January 1, 2022, to July 31, 2023.

**Exposures:**

Informed consent for a therapeutic childhood cancer clinical trial.

**Main Outcomes and Measures:**

The primary outcome of interest was IC comprehension and its associations with SDOH (marital status, language, educational attainment, employment, insurance type, socioeconomic status, and health literacy) and sociocontextual factors (ethnicity, satisfaction with informed consent, and cancer type).

**Results:**

Of 223 parents, 172 (77.1%) were aged 18 to 44 years, 111 (49.8%) were Hispanic, 152 (68.2%) were women, and 163 (73.1%) were married. In terms of race, 2 (0.9%) were American Indian or Alaska Native, 22 (9.9%) were Asian or Pacific Islander, 8 (3.6%) were Black, 149 (66.8%) were White, and 42 (18.8%) were more than 1 race. In multivariable linear mixed-effects analyses, limited vs adequate health literacy was associated with lower comprehension of informed consent overall (mean [SD], 68.28 [11.81] vs 79.24 [11.77]; β estimate, −9.02 [95% CI, −12.0 to −6.07]; *P* < .001) and with lower comprehension of the purpose, procedures, and randomization (mean [SD], 65.00 [12.64] vs 76.14 [11.53]; β estimate, −7.87 [95% CI, −10.9 to −4.85]; *P* < .001); risks and benefits (mean [SD], 62.84 [20.24] vs 73.14 [20.86]; β estimate, −10.1 [95% CI, −15.6 to −4.59]; *P* < .001); alternatives (mean [SD], 54.27 [43.18] vs 82.98 [34.24]; β estimate, −14.3 [95% CI, −26.1 to −2.62]; *P*  .02); and voluntariness (mean [SD], 76.52 [24.33] vs 95.39 [13.89]; β estimate, −9.14 [95% CI, −14.9 to −3.44]; *P* = .002) domains. Use of Spanish vs English language for medical communication was associated with lower comprehension overall (mean [SD], 66.45 [12.32] vs 77.25 [12.18]; β estimate, −5.30 [95% CI, −9.27 to −1.34]; *P* = .01) and with lower comprehension of the purpose, procedures, and randomization (mean [SD], 63.33 [11.98] vs 74.07 [12.52]; β estimate, −4.33 [95% CI, −8.43 to −0.23]; *P* = .04) and voluntariness (mean [SD], 70.83 [24.02] vs 92.54 [17.27]; β estimate, −9.69 [95% CI, −16.8 to −2.56]; *P* = .009) domains.

**Conclusions and Relevance:**

In this cross-sectional study including parents of children with newly diagnosed cancer who provided IC for their child’s participation in a therapeutic clinical trial, limited health literacy and use of Spanish language for medical communication were associated with lower comprehension of IC. These findings suggest that, in this setting, parents with limited health literacy or those who use Spanish language for medical communication may not fully comprehend IC and therefore may not make truly informed decisions. These findings support the investigation of interventions, across pediatric disciplines, tailored to the participant’s language and health literacy level to improve IC comprehension, particularly in racial and ethnic minority populations.

## Key Points

**Question:**

Are social determinants of health and sociocontextual factors associated with informed consent (IC) comprehension in therapeutic childhood cancer clinical trials?

**Findings:**

In this cross-sectional study that included 223 parents of children with newly diagnosed cancer, limited health literacy and use of Spanish language for medical communication were associated with lower comprehension of IC.

**Meaning:**

These findings suggest that parents who have limited health literacy and who use Spanish language for medical communication may not fully comprehend IC and may not be able to make informed decisions for their children.

## Teaser

This cross-sectional study investigates associations of social determinants of health (SDOH) and sociocontextual factors with informed consent comprehension in among parents enrolling their child with newly diagnosed cancer into a therapeutic clinical trial.

## Introduction

Before children can participate in a clinical trial, their parents or guardians must provide informed consent (IC), which is a fundamental ethical obligation in health care. Key tenets of valid IC include information disclosure, competent decision-making, adequate comprehension, and voluntariness.^1,2^ However, numerous barriers to IC in the oncology setting include the use of medical jargon, misunderstandings about clinical trials, tremendous emotional distress surrounding the initial cancer diagnosis, and the complexity and length of the IC forms.^3,4,5,6,7,8^ These barriers may be exacerbated by adverse social determinants of health (SDOH), such as low educational attainment, low socioeconomic status (SES), use of a language other than English, and limited health literacy.^5,9,10,11^ Health literacy is the ability to access and comprehend health information to successfully navigate health care.^12,13^ Limited health literacy is associated with negative health outcomes in children and adults, such as increased rates of hospitalization and lower adherence to medication regimens and preventive care.^13^

Research investigating the role of SDOH in IC comprehension in childhood cancer clinical trials in diverse populations is lacking,^9,10,12^ particularly among Hispanic individuals and those who use a language other than English for medical communication. To address these gaps, we conducted a cross-sectional study to investigate associations of SDOH and sociocontextual factors (eg, ethnicity, satisfaction with IC, and cancer type) with IC comprehension among parents enrolling their child with newly diagnosed cancer in a therapeutic clinical trial. We hypothesized that parents of Hispanic ethnicity, those who use Spanish language for medical communication, or with limited health literacy would have lower IC comprehension.

## Methods

### Study Participants

Our cross-sectional study included parents and legal guardians of children with newly diagnosed cancer at Rady Children’s Hospital San Diego (RCHSD), a large quaternary academic pediatric hospital that serves more than 1 million children and has a large Hispanic representation (45%). This study followed the Strengthening the Reporting of Observational Studies in Epidemiology (STROBE) reporting guideline for cross-sectional studies, which consists of a checklist of 22 items that are considered essential for good reporting of observational studies. The institutional review board for the University of California, San Diego and RCHSD approved this study. Eligible individuals were approached, and voluntary written IC was obtained from participants after a full description of study procedures, per the Common Rule. Language used for medical communication was assessed during recruitment. No one received compensation or incentive for study participation.

Children aged 0 to 17 years diagnosed with cancer between October 1, 2014, and March 31, 2021, were identified from RCHSD’s cancer registry. Parents were eligible to participate if (1) their child was eligible for a therapeutic clinical trial, (2) they had consented to participate in the therapeutic clinical trial within the previous week, and (3) they understood written and spoken English or Spanish. We excluded parents who had given IC for any therapeutic clinical trial in the past (as these individuals likely had a better understanding of IC due to prior exposure), who had a child with second malignant neoplasm or relapsed disease, or who had a child previously diagnosed with cancer at an outside institution. Standard of care IC for the therapeutic clinical trial was conducted by the treating oncologist in the language used by the parent for medical communication and, if the clinician was not fluent in that language, with a medically trained interpreter either in person (preferred method) or via video conference. Informed consent documents for therapeutic clinical trials at RCHSD were available in English and Spanish.

### Study Procedures

Our primary outcome was objective comprehension of the IC and related domains (purpose, procedures, and randomization; risks and benefits; alternatives; and voluntariness) and their associations with SDOH (marital status, language used for medical communication [verbal and written], educational attainment, employment, insurance type, SES, and health literacy) and sociocontextual factors (ethnicity, satisfaction with IC, and cancer type). Participants were approached in inpatient and outpatient hospital settings and completed questionnaires within 1 week of the IC discussion for the therapeutic clinical trial. Questionnaire items were related to IC comprehension, sociodemographic characteristics, health literacy, and satisfaction with IC. Questionnaires were available in English and Spanish and administered by bilingual (English and Spanish) and bicultural (Anglo and Hispanic) research staff (including P.A. and B.P.P.).^14,15,16,17^ All questionnaire questions were written with a low level of complexity and high readability.^13,15^ Both parents (if available) were invited to participate in the study, and they completed the questionnaires independently. We collected data from the medical record, including patient’s age, sex, and cancer type.

### Study Measures

To assess objective comprehension of the basic elements of IC, we used the 20-item brief, reliable, and valid Quality of Informed Consent (QuIC) instrument.^18^ Its domains are based on the IC guidelines from the US Department of Health and Human Services. We analyzed overall IC comprehension and comprehension of domains related to purpose, procedures, and randomization; risks and benefits; alternatives; and voluntariness. Scores range from 0 to 100; higher scores are indicative of increased comprehension. The QuIC has been validated in adults with cancer and used among parents of children with cancer.^19,20^ The SDOH were assessed using a sociodemographic questionnaire,^12^ which included parental age, self-reported race and ethnicity (categorized per US Census Bureau),^21^ marital status, language used for medical communication (verbal and written), educational attainment, employment status and occupation, and insurance type. Socioeconomic status was calculated using the Hollingshead index (marital status, retired or employed status, educational attainment, and occupational prestige).^22^ The 6-item Newest Vital Sign (NVS) evaluated health literacy based on interpretation of a nutrition label.^15^ Scores of 0 to 3 indicate limited health literacy; 4 to 6, adequate health literacy. The NVS has been validated in other disciplines^23^ and used widely, including among parents who make health decisions for their children.^12,24,25,26^ The 7-item satisfaction questionnaire CCG-S9901 has been used in childhood cancer^8,27^ and assessed perceptions of overall satisfaction with IC discussions, clarity of explanations, quantity of information, and utility of the IC. Higher scores indicate greater satisfaction. The NVS has been validated in English and Spanish.^15^ For instruments not validated in Spanish (the QuIC, CCG-S9901, and sociodemographic questionnaire), we used the Brislin method, which compares back-translated documents with the source language and is used extensively in cross-cultural research.^28,29,30^

### Statistical Analysis

Continuous (eg, SES) and categorical variables (eg, health literacy) were summarized by mean (SD) and count (percentage), respectively. The primary outcome of interest was overall comprehension of IC, and the secondary outcomes were comprehension of related IC domains. The association of outcomes with SDOH and sociocontextual factors (ethnicity, satisfaction with IC, and cancer type) was assessed using a linear mixed-effects model. A random intercept structure was included to account for the cluster effect of parents and guardians from the same family. A parsimonious multivariable linear mixed-effects model was fit with IC comprehension and related domains as the outcome variables. Hispanic ethnicity was the primary covariate of interest and was forced into the multivariable model regardless of its significance level. The SDOH and contextual factors were included in the initial multivariable model only if they were significant in univariable models with *P <* .20 as the cut point. Backward elimination was used to remove insignificant variables from the model until all variables were at *P <* .10. Coefficient estimates (β) and 95% CIs were reported. The threshold for statistical significance for all statistical analyses was 2 sided, and significant associations were defined as *P* ≤ .05. Sample size for enrollment was based on our a priori power calculation to achieve 80% power at the joint 2-sided *P* ≤ .05 significance level to detect a significant Cohen effect size in IC comprehension of 0.31. Missing data were minimized by intensive training of the research staff in techniques of clarifying answers and checking questionnaires while participants were on site. In case of missing data, the missing pattern was examined, and appropriate data analytic techniques were used. All analyses were conducted from January 1, 2022, to July 31, 2023, using statistical software program R, version 4.1.2 (R Project for Statistical Computing).

## Results

### Participant Characteristics

In total, 885 patients aged 0 to 17 years were diagnosed with cancer during the study period, and 309 parents met inclusion criteria for this study and were prospectively approached for enrollment. Of these, 53 eligible parents (17.2%) declined to participate, and 33 enrolled participants (10.7%) opted out of the study (Figure). Reasons for declining or opting out included having no time to complete questionnaires (n = 55), feeling overwhelmed (n = 27), or opting out from the therapeutic clinical trial after enrollment (n = 4). Demographic characteristics of individuals who were not eligible or who declined or opted out of participation in our study were similar to those of participants.

**Figure.**
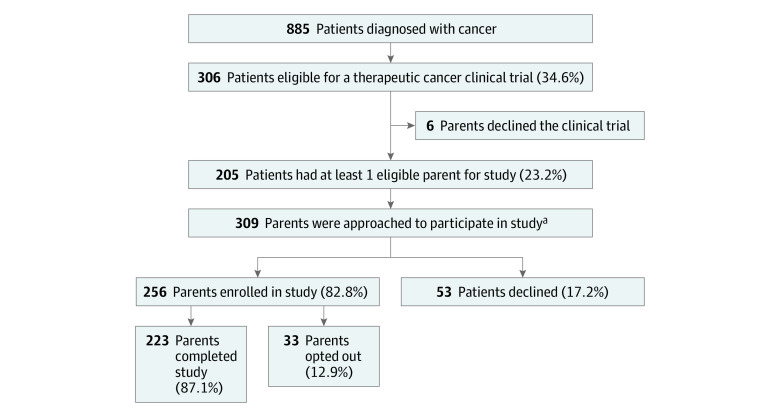
Study Flow Diagram ^a^Of the potential 410 eligible parents, only 309 were approached.

The final sample was representative of the population in the RCHSD catchment area and comprised 223 participants (172 [77.1%] aged 18-44 years old vs 51 [22.9%] 45 years or older), including 45 parental couples. Half of the participants were Hispanic (111 [49.8%] vs 112 [50.2%] non-Hispanic); in terms of race, 2 (0.9%) were American Indian or Alaska Native, 22 (9.9%) were Asian or Pacific Islander, 8 (3.6%) were Black, 149 (66.8%) were White, and 42 (18.8%) were more than 1 race. Most participants were women (152 [68.2%] vs 71 [31.8%] men), aged 18 to 44 years (172 [77.1%]), and married (163 [73.1%]). Among Hispanic individuals, 42 (37.8%) used Spanish language for medical communication. Among the 178 children, most were younger than 10 years (114 [64.0%]) and diagnosed with a hematological malignant neoplasm (141 [79.2%]). Sociodemographic characteristics and outcome measures are described in Table 1 and Table 2.

**Table 1.** Baseline Characteristics of Study Participants

Characteristic	Parent group^a^		
	Non-Hispanic (n = 112 [50.2])	Hispanic (n = 111 [49.8])	All (N = 223)
Age, y			
18-44	78 (69.6)	94 (84.7)	172 (77.1)
≥45	34 (30.4)	17 (15.3)	51 (22.9)
Sex			
Women	70 (62.5)	82 (73.9)	152 (68.2)
Men	42 (37.5)	29 (26.1)	71 (31.8)
Race			
American Indian or Alaska Native	1 (0.9)	1 (0.9)	2 (0.9)
Asian or Pacific Islander	21 (18.8)	1 (0.9)	22 (9.9)
Black or African American	7 (6.3)	1 (0.9)	8 (3.6)
White	77 (68.8)	72 (64.9)	149 (66.8)
>1	6 (5.4)	36 (32.4)	42 (18.8)
Marital status			
Unmarried^b^	20 (17.9)	40 (36.0)	60 (26.9)
Married	92 (82.1)	71 (64.0)	163 (73.1)
Language used for medical communication			
Spanish	0	42 (37.8)	42 (18.8)
English	112 (100)	69 (62.2)	181 (81.2)
Level of education			
High school or less	6 (5.4)	54 (48.6)	60 (26.9)
Some college	38 (33.9)	31 (27.9)	69 (30.9)
College	68 (60.7)	26 (23.4)	94 (42.2)
Employment status^c^			
Informally employed or unemployed	40 (35.7)	58 (55.2)	98 (45.2)
Employed	72 (64.3)	47 (44.8)	119 (54.8)
Insurance type			
Public	40 (35.7)	84 (75.7)	124 (55.6)
Private	72 (64.3)	27 (24.3)	99 (44.4)
SES Hollingstead index score, mean (SD)^d^	44.16 (13.43)	30.87 (14.86)	37.57 (15.62)
NVS health literacy score			
Limited (0-3)	18 (16.1)	64 (57.7)	82 (36.8)
Adequate (4-6)	94 (83.9)	47 (42.3)	141 (63.2)
Satisfaction questionnaire CCG-S9901 score, mean (SD)^e^	17.95 (2.53)	17.23 (3.15)	17.59 (2.88)
Child’s age, y^f^			
<10	56 (65.1)	58 (63.0)	114 (64.0)
≥10	30 (34.9)	34 (37.0)	64 (36.0)
Cancer type^f^			
Solid	17 (19.8)	20 (21.7)	37 (20.8)
Hematological	69 (80.2)	72 (78.3)	141 (79.2)

Abbreviations: NVS, Newest Vital Sign; SES, socioeconomic status.

^a^ Unless otherwise indicated, data are expressed as No. (%) of parents. Demographic characteristics did not vary significantly by parental study participation vs decline.

^b^ Includes single, divorced, widowed, and separated.

^c^ Six parents selected “prefer not to say.”

^d^ One parent selected “prefer not to say.” Scores range from 8 to 66, with higher scores indicating higher SES.

^e^ Scores range from 7 to 21, with higher scores indicating greater satisfaction.

^f^ Includes 178 children (86 non-Hispanic and 92 Hispanic).

**Table 2.** Outcome Measures of Study Participants^a^

Outcome measures	Participants		
	Non-Hispanic (n = 112)	Hispanic (n = 111)	All (N = 223)
Overall IC comprehension^b^	78.64 (13.07)	71.75 (11.80)	75.21 (12.89)
IC comprehension domain^b^			
Purposes, procedures, and randomization	75.68 (12.71)	68.38 (12.47)	72.04 (13.09)
Risks and benefits	72.10 (21.68)	66.58 (20.39)	69.35 (21.18)
Alternatives	79.02 (37.75)	65.77 (41.55)	72.42 (40.15)
Voluntariness	93.75 (16.27)	83.11 (22.90)	88.45 (20.51)

Abbreviation: IC, informed consent.

^a^ Data are presented as mean (SD). Demographics did not vary significantly by parental study participation vs decline.

^b^ Scores range from 0 to 100, with higher scores indicating higher comprehension.

### Overall IC Comprehension 

Overall IC comprehension was assessed by SDOH and sociocontextual factors. In multivariable analysis, unmarried vs married status (mean [SD], 69.89 [11.84] vs 77.18 [12.74]; β estimate, −4.51 [95% CI, −7.73 to −1.28]; *P* = .007), Spanish vs English language used for medical communication (mean [SD], 66.45 [12.32] vs 77.25 [12.18]; β estimate, −5.30 [95% CI, −9.27 to −1.34]; *P* = .01), limited vs adequate health literacy (mean [SD], 68.28 [11.81] vs 79.24 [11.77]; β estimate, −9.02 [95% CI, −12.0 to −6.07]; *P* < .001), and lower satisfaction with IC (mean [SD], 0.89 [95% CI, 0.46-1.34]; *P* < .001) were associated with lower comprehension (Table 3 and eTable 1 in Supplement 1).

**Table 3.** Comprehension of IC Outcomes Associated With Social Determinants of Health and Sociocontextual Factors of Parents^a^

Variable	Estimate, β (SE) [95% CI]^b^	*P* value^c^
Comprehension of IC overall		
Ethnicity (Hispanic vs non-Hispanic)	−0.002 (1.67) [−3.24 to 3.24]	>.99
Marital status (unmarried vs married)	−4.51 (1.66) [−7.73 to −1.28]	.007
Language used for medical communication (Spanish vs English)		
	−5.30 (2.04) [−9.27 to −1.34]	.01
Health literacy (limited vs adequate)	−9.02 (1.51) [−12.0. to −6.07]	<.001
Satisfaction with IC	0.89 (0.22) [0.46 to 1.34]	<.001
Comprehension of purpose, procedures, and randomization		
Parental age (18-44 vs ≥45 y)	4.07 (1.71) [0.77 to 7.38]	.02
Ethnicity (Hispanic vs non-Hispanic)	−3.11 (1.74) [−6.57 to 0.34]	.08
Marital status (unmarried vs married)	−3.16 (1.75) [−6.5 4 to 0.23]	.07
Language used for medical communication (Spanish vs English)		
	−4.33 (2.11) [−8.43 to −0.23]	.04
Health literacy (limited vs adequate)	−7.87 (1.55) [−10.9 to −4.85]	<.001
Satisfaction with IC	0.63 (0.23) [0.18 to 1.10]	.006
Comprehension of risks and benefits		
Ethnicity (Hispanic vs non-Hispanic)	1.71 (3.02) [−4.34 to 7.72]	.57
Health literacy (limited vs adequate)	−10.1 (2.81) [−15.6 to −4.59]	<.001
Cancer type (solid vs hematological)	−8.32 (3.67) [−15.5 to −1.15]	.03
Comprehension of alternatives		
Parental age (18-44 vs ≥45 y)	11.4 (5.98) [−0.21 to 23.1]	.06
Ethnicity (Hispanic vs non-Hispanic)	6.17 (5.93) [−5.31 to 17.7]	.30
Socioeconomic status	0.47 (0.20) [0.08 to 0.85]	.02
Health literacy (limited vs adequate)	−14.3 (5.91) [−26.1 to −2.62]	.02
Satisfaction with IC	2.62 (0.91) [0.8 5to 4.38]	.004

Abbreviation: IC, informed consent.

^a^ Sociocontextual factors include ethnicity, satisfaction with IC, and cancer type.

^b^ Calculated from linear mixed effects model.

^c^ Significant variables in multivariable model are from the parsimonious multivariable models after backward elimination.

### Purpose, Procedures, and Randomization Domain 

We assessed comprehension of the purpose, procedures, and randomization process. In multivariable analysis, older vs younger parents (mean [SD], 70.31 [11.82] vs 72.56 [13.43]; β estimate, 4.07 [95% CI, 0.77-7.38]; *P* = .02), Spanish vs English language used for medical communication (mean [SD], 63.33 [11.98] vs 74.07 [12.52]; β estimate, −4.33 [95% CI, −8.43 to −0.23]; *P* = .04), limited vs adequate health literacy (mean [SD], 65.00 [12.64] vs 76.14 [11.53]; β estimate, −7.87 [95% CI, −10.9 to −4.85]; *P* < .001), and lower satisfaction with IC (β estimate, 0.63 [95% CI, 0.18-1.10]; *P* = .006) were associated with lower comprehension of the domain (Table 3 and eTable 2 in Supplement 1).

### Risks and Benefits Domain 

We assessed comprehension of risks and benefits. In multivariable analysis, limited vs adequate health literacy (mean [SD], 62.84 [20.24] vs 73.14 [20.86]; β estimate, −10.1 [95% CI, −15.6 to −4.59]; *P* < .001) and solid vs hematological tumor type (mean [SD], 61.98 [19.6] vs 71.37 [21.24]; β estimate, −8.32 [95% CI, −15.5 to −1.15]; *P* = .03) were associated with lower comprehension of the domain (Table 3 and eTable 3 in Supplement 1).

### Alternatives Domain

We assessed comprehension of alternative treatment options. In multivariable analysis, lower SES (β estimate, 0.47 [95% CI, 0.08-0.85]; *P* = .03), limited vs adequate health literacy (mean [SD], 54.27 [43.18] vs 82.98 [34.24]; β estimate, −14.3 [95% CI, −26.1 to −2.62]; *P* = .02) and lower satisfaction with IC (β estimate, 2.62 [95% CI, 0.85-4.38]; *P* = .004) were associated with lower comprehension of alternatives (Table 3 and eTable 4 in Supplement 1).

### Voluntariness Domain 

We assessed comprehension of voluntariness. In multivariable analysis, Spanish vs English language used for medical communication (mean [SD], 70.83 [24.02] vs 92.54 [17.27]; β estimate, −9.69 [95% CI, −16.8 to −2.56]; *P* = .009), lower SES (β estimate, 0.22 [95% CI, 0.05-0.40]; *P* = .02), and limited vs adequate health literacy (mean [SD], 76.52 [24.33] vs 95.39 [13.89]; β estimate, −9.14 [95% CI, −14.9 to −3.44]; *P* = .002) were associated with lower comprehension of voluntariness (eTable 5 in Supplement 1).

## Discussion

This cross-sectional study investigated SDOH and sociocontextual factors associated with IC comprehension among parents who had consented to their child’s participation in a therapeutic clinical trial for cancer. One of our key findings was that, on multivariable analysis, limited health literacy was consistently associated with lower overall comprehension of IC and all IC comprehension domains analyzed (purpose, procedures, and randomization; risks and benefits; alternatives; and voluntariness)*.* Health literacy is closely linked to literacy and entails the individual’s knowledge and skills to access, comprehend, and apply health information and thereby make informed health decisions.^13^ A review of over 100 IC documents for clinical trials found an average grade 12 reading level, suggesting that limited literacy poses a barrier to IC comprehension.^31^ Health literacy–concordant medical communication is critical for patients to successfully navigate the complexities of clinical trials.^12,31^ We previously reported that limited health literacy is associated with decreased perception of voluntariness during IC for clinical trials in leukemia.^12^ In the present study, we found that limited health literacy was associated with lower comprehension of IC voluntariness, suggesting that parents may not understand the key concept of providing voluntary IC.

Consistent with our findings, past research has revealed challenges with parental comprehension of essential elements of IC. Knifed et al^32^ reported that 33% of parents did not recall any risks associated with the clinical trial or recalled only 1 to 4 risks, which tended to be generic. Many parents had difficulty understanding study procedures, randomization, and alternatives to the clinical trial.^3,4,8,32^ One study^33^ showed that only 32% of parents had an understanding of the purpose behind the clinical trial, and most of these parents were non-Hispanic White individuals with higher SES. Therefore, implementation of strategies to enhance IC comprehension is critical.

There is a dramatic underrepresentation of children of Hispanic and Spanish-speaking parents participating in clinical trials for childhood cancer.^34,35^ Our study included a significant proportion of Hispanic parents (49.8%) and individuals who used Spanish for medical communication (37.8% of Hispanic parents), allowing assessment of outcomes in these understudied populations. Use of Spanish language for medical communication was associated with lower overall IC comprehension and lower comprehension of the purpose, procedures, and randomization and voluntariness domains, highlighting the importance of language-concordant IC delivery to ensure IC comprehension. Among Hispanic cultural norms, respect may affect IC comprehension,^36^ as Hispanic parents may be hesitant to ask clarifying questions to the clinician. In a qualitative study, parents who communicated in a language other than English were less likely to ask questions compared with those who used English when presented with IC forms for cancer clinical trials.^9^ Similarly to culture, language discordance plays an important role in the delivery of IC. Discussions of IC have been reported to be about equal in duration for both participants who use an interpreter and those who use English, which implies that clinicians may shorten the IC discussion for individuals who do not use English for medical communication.^9^ Also, studies have shown that parents who use a language other than English had greater difficulty understanding “their right to withdraw from research studies” or randomization,^9^ even 6 months after their child was enrolled in a clinical trial.^37^ Our finding of lower comprehension of the purpose, procedures, and randomization domain being associated with use of Spanish language for medical communication is congruent with the literature.^9,10^

We also found that unmarried marital status, older parental age, and lower SES were associated with lower IC comprehension. Associations between marital status and IC are not well delineated. Discussion among partners or “teach back” (where individuals explain health information in their own words^38^) to each other may improve IC comprehension. Moreover, unmarried parents and those with lower SES may be burdened by other responsibilities that detract from focus and time spent understanding IC, or they may be more likely to come from underserved backgrounds and sociocultural contexts may influence their comprehension.^10,11^ Our results support identifying unmarried, older parents and those with lower SES who may need additional support. These findings contribute to the scant literature on adverse SDOH and associations with outcomes in parents of children with cancer.^10,12,35^

In our study, less satisfaction with IC was associated with lower IC comprehension. This association is not well understood. In contrast to prior research that reported that parents who did not comprehend basic elements of IC were still satisfied with the IC process,^4,8^ our findings suggest that there may be a direct association between overall perceived satisfaction with IC and IC comprehension that warrants further investigation. Based on our results, we postulate that parental satisfaction with IC is a proxy for effective IC delivery by the clinician, and effective delivery is essential for comprehension.

Obtaining IC in the oncology setting poses unique challenges. With the devastating news of a childhood cancer diagnosis, the shock experienced by parents can affect IC comprehension.^11,27^ Additionally, the time-sensitive nature of starting cancer treatment creates a high-stakes environment that is not conducive to comprehension.^39^ The length of IC documents has also increased by 10-fold within a span of 3 decades.^31,39^ Additionally, there is a reported lack of formal training for clinicians to successfully deliver IC.^11^

To improve IC comprehension for clinical trials in childhood cancer, it is imperative that IC discussions are tailored to the language, health literacy, and cultural needs of families. Furthermore, using in-person interpreters facilitates a better understanding of IC content in different languages.^40,41^ For parents who may hesitate to ask questions, engaging them in decision-making and assessing their understanding using techniques such as the teach back method can be helpful.^36,39^ Moreover, a tiered, staged IC approach^42^ and interventions using decision aids and patient navigation may improve IC comprehension and decision-making.^43,44^ Finally, strategies at the clinician and health care system levels are also urgently needed.^11,35,45^

### Limitations

This study has some limitations. Although our overall sample was representative of the population we serve in our catchment area, it was limited by the inclusion of more mothers than fathers, a small proportion of Asian or Pacific Islander and Black individuals, and the inclusion only of English- and Spanish-speaking parents. Hispanic participants were primarily from Mexico, and although individuals of Mexican descent are the largest Hispanic group (62%) in the US,^46^ this may constrain the generalizability of our findings to other Latin American subgroups. Our analysis accounted for the cluster effect of parents from the same family; however, we did not have sufficient parental couples to perform paired cross-parent comparisons, thereby potentially causing bias or precluding comparisons among subgroups. Future research should include a larger and more diverse sample to allow for subgroup comparisons and sensitivity analyses. Our sample included mostly parents of patients with hematological malignant neoplasms, which are the most common pediatric cancer; nevertheless, this may limit the applicability of our findings to parents of children with solid tumors. Of note, during the study period, there were more therapeutic clinical trials open for enrollment for hematological than for solid tumors. Additionally, if the participant completed the IC comprehension questionnaire prior to completing the satisfaction with IC questionnaire, this could have affected satisfaction scores. Furthermore, we did not survey those who declined to participate in the therapeutic clinical trial to determine reasons for declining. Thus, our findings may not represent those who have baseline hesitancy about research participation. Another limitation is that participants with lower health literacy might have had greater difficulty understanding the questionnaires as well as the IC, which might have contributed to the observed differences by health literacy. We performed only quantitative measurements and did not assess the training of the clinician or the interpreter delivering the IC, the quality of the information delivered, or the duration or location (wards vs intensive care unit) of the IC discussion, preventing the depth of understanding of the complexity of IC, such as the role of patient-clinician communication and the clinician-level factors that could affect comprehension independently.^11,47,48,49^ We acknowledge that we did not conduct a comprehensive assessment of all domains of SDOH and analyzed mostly those at the individual level, limiting the understanding of the role of community and neighborhood factors. Future research should include inclusive evaluations of SDOH, characterization of cultural and linguistic patient-clinician concordance, theory-guided qualitative studies to further understanding of barriers and facilitators to adequate IC, assessments of longitudinal retention of the information in the IC form, and evaluation of the role of clinician training in cultural and health literacy competencies to enhance IC delivery. Last, inclusion of sociocontextual factors in the same multivariable model including SDOH may obscure or exacerbate the associations of variables with the outcomes.

## Conclusions

In this cross-sectional study, although IC comprehension is conceptually complex, the significant associations we found for limited health literacy and Spanish used for medical communication with low IC comprehension highlight the critical need to timely identify and support individuals with adverse SDOH, including limited health literacy and/or use of a language other than English during clinical trial recruitment. Future research should be conducted to ensure that all clinical trial participation is voluntary and that all elements of IC are fully comprehended by the participant or proxy decision-maker. Our findings support the investigation across pediatric disciplines of the potential role of culturally, linguistically, and health literacy–concordant interventions to improve IC comprehension and decision-making and ensure more equitable research participation in racial and ethnic minority populations.

## Notes

Supplement 1. **eTable 1.** Overall IC Comprehension Associated With Social Determinants of Health (SDOH) and Sociocontextual Factors **eTable 2.** Comprehension of Purpose, Procedures, and Randomization Associated With Social Determinants of Health (SDOH) and Sociocontextual Factors **eTable 3.** Comprehension of Risks and Benefits Associated With Social Determinants of Health (SDOH) and Sociocontextual Factors **eTable 4.** Comprehension of Alternatives Associated With Social Determinants of Health (SDOH) and Sociocontextual Factors **eTable 5.** Comprehension of Voluntariness Associated With Social Determinants of Health (SDOH) and Sociocontextual Factors

Supplement 2.
Data Sharing Statement
