# Supplementary material for: Social Determinants of Health and Informed Consent Comprehension for Pediatric Cancer Clinical Trials
Source: JAMA Netw Open. 2023 Dec 11;6(12):e2346858. doi: 10.1001/jamanetworkopen.2023.46858 (PMC10714248; doi:10.1001/jamanetworkopen.2023.46858)
Supplement: Supplement 1. — eTable 1. Overall IC Comprehension Associated With Social Determinants of Health (SDOH) and Sociocontextual Factors eTable 2. Comprehension of Purpose, Procedures, and Randomization Associated With Social Determinants of Health (SDOH) and Sociocontextual Factors eTable 3. Comprehension of Risks and Benefits Associated With Social Determinants of Health (SDOH) and Sociocontextual Factors eTable 4. Comprehension of Alternatives Associated With Social Determinants of Health (SDOH) and Sociocontextual Factors eTable 5. Comprehension of Voluntariness Associated With Social Determinants of Health (SDOH) and Sociocontextual Factors [file jamanetwopen-e2346858-s001.pdf]

Supplemental Online Content

Aristizabal P, Nataraj S, Ma AK, et al. Social determinants of health and informed consent comprehension for pediatric cancer clinical trials. *JAMA Netw Open*. 2023;6(12):e2346858. doi:10.1001/jamanetworkopen.2023.46858

eTable 1. Overall IC Comprehension Associated With Social Determinants of Health (SDOH) and Sociocontextual Factors

eTable 2. Comprehension of Purpose, Procedures, and Randomization Associated With Social Determinants of Health (SDOH) and Sociocontextual Factors

eTable 3. Comprehension of Risks and Benefits Associated With Social Determinants of Health (SDOH) and Sociocontextual Factors

eTable 4. Comprehension of Alternatives Associated With Social Determinants of Health (SDOH) and Sociocontextual Factors

eTable 5. Comprehension of Voluntariness Associated With Social Determinants of Health (SDOH) and Sociocontextual Factors

This supplemental material has been provided by the authors to give readers additional information about their work.

eTable 1. Overall IC Comprehension Associated With Social Determinants of Health (SDOH) and Sociocontextual Factors[ab]

	Univariable		Multivariable[c]	
**Variable**	**Estimate, β [95% CI][d]**	***P***	**Estimate, β [95% CI]**	***P***
**Parental Age** (Ref. ≥45)				
18-44	−0.01 [−4.03 to 4.02]	1.00		
**Parental Ethnicity** (Ref. Non-Hispanic)				
Hispanic	-6.31 [-9.72 to -2.89]	<0.001	-0.002 [-3.24 to 3.24]	1.00
**Marital Status** (Ref. Married)				
Unmarried[e]	-7.25 [-11.1 to -3.38]	<0.001	-4.51 [-7.73 to -1.28]	0.01
**Preferred Language for Medical Communication** (Ref. English)				
Spanish	-10.4 [-14.6 to -6.26]	<0.001	-5.30 [-9.27 to -1.34]	0.01
**Level of Education** (Ref. College)				
High School or Less	-8.28 [-12.2 to -4.33]	<0.001		
Some College	-0.91 [-4.47 to 2.69]	0.61		
**Employment Status** (Ref. Employed)				
Informally Employed/ Unemployed	-1.33 [-4.41 to 1.73]	0.39		
**Insurance Type** (Ref. Private)				
Public	-4.60 [-8.24 to -0.95]	0.01		
**Socioeconomic Status[f]**	0.24 [0.13 to 0.36]	<0.001		
**Health Literacy[g]** (Ref. Adequate)				
Limited	-10.7[-13.6 to -7.74]	<0.001	-9.02 [-12.0. to -6.07]	<0.001
**Satisfaction with IC[h]**	1.03 [0.52 to 1.54]	<0.001	0.89 [0.46 to 1.34]	<0.001
**Cancer Type** (Ref. Hematologic)				
Solid	-2.53 [-7.06 to 2.02]	0.27		

Abbreviations: IC, Informed Consent; SDOH, Social Determinants of Health; CI, Confidence Interval.
[a]Overall IC comprehension score (range 0-100).
[b]Ethnicity, Satisfaction with informed consent, Cancer type.
[c]Significant variables in multivariable model are from the parsimonious multivariable models after backward elimination.
[d]Coefficient estimate, 95% CI and *P-value*, from linear mixed effects model.
[e]Unmarried includes single, divorced, widowed, and separated.
[f]Socioeconomic Status score (range 8-66).
[g]Health literacy adequate is defined as score (range 4-6).
[h]Satisfaction with IC score (range 7-21).

© 2023 Aristizabal P et al. *JAMA Network Open.*

eTable 2. Comprehension of Purpose, Procedures, and Randomization Associated With Social Determinants of Health (SDOH) and Sociocontextual Factors[b]

	Univariable		Multivariable[c]	
**Variable**	**Estimate, β [95% CI]**[d]	***P***	**Estimate, β [95% CI]**	***P***
**Parental Age** (Ref. ≥45)				
18-44	2.65 [-1.33 to 6.64]	0.19	4.07 [0.77 to 7.38]	0.02
**Parental Ethnicity** (Ref. non-Hispanic)				
Hispanic	-7.95 [-11.4 to -4.53]	<0.001	-3.11 [-6.57 to 0.34]	0.08
**Marital Status** (Ref. Married)				
Unmarried[e]	-5.55 [-9.55 to -1.55]	0.007	-3.16 [-6.5 4 to 0.23]	0.072
**Preferred Language for Medical Communication** (Ref. English)				
Spanish	-10.1 [-14.3 to -5.87]	<0.001	-4.33 [-8.43 to -0.23]	0.04
**Level of Education** (Ref. College)				
High School or Less	-8.71 [-12.7 to -4.77]	<0.001		
Some College	-0.98 [-4.57 to 2.76]	0.58		
**Employment Status** (Ref. Employed)				
Informally Employed/ Unemployed	-0.87 [-3.89 to 2.13]	0.57		
**Insurance Type** (Ref. Private)				
Public	-3.78 [-7.55 to -0.01]	0.05		
**Socioeconomic Status**[f]	0.19 [0.07 to 0.31]	0.002		
**Health Literacy**[g] (Ref. Adequate)				
Limited	-9.99 [-13.1 to -6.98]	<0.001	-7.87 [-10.9 to -4.85]	<0.001
**Satisfaction with IC**[h]	0.73 [0.22 to 1.25]	0.005	0.63 [0.18 to 1.10]	0.006
**Cancer Type** (Ref. Hematologic)				
Solid	-0.07 [-4.78 to 4.65]	0.98		

Abbreviations: IC, Informed Consent; SDOH, Social Determinants of Health; CI, Confidence Interval.
[a]Comprehension of Purposes and procedures and randomization domain score (range 0-100).
[b]Ethnicity, Satisfaction with informed consent, Cancer type.
[c]Significant variables in multivariable model are from the parsimonious multivariable models after backward elimination.
[d]Coefficient estimate, 95% CI and *P-value*, from linear mixed effects model.
[e]Unmarried includes single, divorced, widowed, and separated.
[f]Socioeconomic Status score (range 8-66).
[g]Health literacy adequate is defined as score (range 4-6).
[h]Satisfaction with IC score (range 7-21).

© 2023 Aristizabal P et al. *JAMA Network Open.*

eTable 3. Comprehension of Risks and Benefits Associated With Social Determinants of Health (SDOH) and Sociocontextual Factors[ab]

Variable	Univariable Estimate, β [95% CI][d]	Univariable *P*	Multivariable[c] Estimate, β [95% CI]	Multivariable[c] *P*
**Parental Age** (Ref. ≥45)				
18-44	-2.98 [-9.48 to 3.51]	0.37		
**Parental Ethnicity** (Ref. Non-Hispanic)				
Hispanic	-2.73 [-8.50 to 3.09]	0.34	1.71 [-4.34 to 7.72]	0.572
**Marital Status** (Ref. Married)				
Unmarried[e]	-6.86 [-13.3 to -0.45]	0.04		
**Preferred Language** for Medical Communication (Ref. English)				
Spanish	-7.55 [-14.6 to -0.52]	0.04		
**Level of Education** (Ref. College)				
High School or Less	-6.24 [-12.8 to -0.26]	0.06		
Some College	-2.74 [-8.53 to 3.01]	0.35		
**Employment Status** (Ref. Employed)				
Informally Employed/ Unemployed	-2.8 (2.5) [-7.7 to 2.1]	0.26		
**Insurance Type** (Ref. Private)				
Public	-3.74 [-9.72 to 2.26]	0.22		
**Socioeconomic Status**[f]	0.28 [0.09 to 0.47]	0.004		
**Health Literacy**[g] (Ref. Adequate)				
Limited	-9.46 [-14.6 to -4.30]	<0.001	-10.1 [-15.6 to -4.59]	<0.001
**Satisfaction with IC**[h]	0.32 [-0.53 to 1.18]	0.46		
**Cancer Type** (Ref. Hematologic)				
Solid	-8.12 [-15.4 to -0.81]	0.03	-8.32 [-15.5 to -1.15]	0.03

Abbreviations: IC, Informed Consent; SDOH, Social Determinants of Health; CI, Confidence Interval.
[a] Comprehension of risks/benefits domain score (range 0-100).
[b] Ethnicity, Satisfaction with informed consent, Cancer type.
[c]Significant variables in multivariable model are from the parsimonious multivariable models after backward elimination
[d]Coefficient estimate, 95% CI and *P-value*, from linear mixed effects model.
[e]Unmarried includes single, divorced, widowed, and separated.
[f]Socioeconomic Status score (range 8-66).
[g]Health literacy adequate is defined as score (range 4-6).
[h]Satisfaction with IC score (range 7-21).

© 2023 Aristizabal P et al. *JAMA Network Open.*

eTable 4. Comprehension of Alternatives Associated With Social Determinants of Health (SDOH) and Sociocontextual Factors[ab]

	Univariable		Multivariable [c]	
**Variable**	**Estimate, β [95% CI][d]**	***P***	**Estimate, β [95% CI]**	***P***
**Parental Age** (Ref. ≥45)				
18-44	9.60 [-3.24 to 22.4]	0.14	11.4 [-0.21 to 23.1]	0.06
**Parental Ethnicity** (Ref. Non-Hispanic)				
Hispanic	-12.1 [-23.0 to -1.13]	0.03	6.17 [-5.31 to 17.7]	0.30
**Marital Status** (Ref. Married)				
Unmarried[e]	-15.0 [-27.4 to -2.73]	0.02		
**Preferred Language for Medical Communication** (Ref. English)				
Spanish	-25.0 [-38.5 to -11.6]	<0.001		
**Level of Education** (Ref. College)				
High School or Less	-23.1 [-35.7 to -10.5]	<0.001		
Some College	-1.37 [-12.9 to 10.2]	0.82		
**Employment Status** (Ref. Employed)				
Informally Employed/ Unemployed	-2.40 [-12.6 to 7.83]	0.644		
**Insurance Type** (Ref. Private)				
Public	-10.3 [-21.6 to -1.14]	0.08		
**Socioeconomic Status[f]**	0.67 [0.32 to 1.02]	<0.001	0.47 [0.08 to 0.85]	0.02
**Health Literacy[g]** (Ref. Adequate)				
Limited	-26.1 [-36.6 to -15.7]	<0.001	-14.3 [-26.1 to -2.62]	0.017
**Satisfaction with IC[h]**	3.49 [1.76 to 5.18]	<0.001	2.62 [0.8 5to 4.38]	0.004
**Cancer Type** (Ref. Hematologic)				
Solid	4.48 [-9.54 to 18.6]	0.53		

Abbreviations: IC, Informed Consent; SDOH, Social Determinants of Health; CI, Confidence Interval.
[a]Comprehension of alternatives domain score (range 0-100).
[b] Ethnicity, Satisfaction with informed consent, Cancer type.
[c]Significant variables in multivariable model are from the parsimonious multivariable models after backward elimination.
[d]Coefficient estimate, 95% CI and *P-value*, from linear mixed effects model.
[e]Unmarried includes single, divorced, widowed, and separated.
[f]Socioeconomic Status score (range 8-66).
[g]Health literacy adequate is defined as score (range 4-6).
[h]Satisfaction with IC score (range 7-21).

© 2023 Aristizabal P et al. *JAMA Network Open.*

eTable 5. Comprehension of Voluntariness Associated With Social Determinants of Health (SDOH) and Sociocontextual Factors[ab]

	Univariable		Multivariable [c]	
**Variable**	**Estimate, β [95% CI][d]**	***P***	**Estimate, β [95% CI]**	***P***
**Parental Age** (Ref. ≥45)				
18-44	1.90 [-4.60 to 8.49]	0.57		
**Parental Ethnicity** (Ref. Non-Hispanic)				
Hispanic	-10.5 [-16.0 to -5.08]	<0.001	3.29 [-2.26 to 8.84]	0.25
**Marital Status** (Ref. Married)				
Unmarried[e]	-4.41 [-10.8 to 1.95]	0.17		
**Preferred Language for Medical Communication** (Ref. English)				
Spanish	-21.4 [-27.8 to -14.9]	<0.001	-9.69 [-16.8 to -2.56]	0.009
**Level of Education** (Ref. College)				
High School or Less	-19.2 [-25.3 to -13.0]	<0.001		
Some College	0.065 [-5.68 to 5.82]	0.98		
**Employment Status** (Ref. Employed)				
Informally Employed/ Unemployed	-8.97 [-14.3 to -3.69]	<0.001		
**Insurance Type** (Ref. Private)				
Public	-11.2 [-16.8 to -5.61]	<0.001		
**Socioeconomic Status[f]**	0.50 [0.34 to 0.67]	<0.001	0.22 [0.05 to 0.40]	0.02
**Health Literacy[g]** (Ref. Adequate)				
Limited	-18.5 [-23.6 to -13.3]	<0.001	-9.14 [-14.9 to -3.44]	0.002
**Satisfaction with IC[i]**	0.11 [-0.79 to 1.01]	0.81		
**Cancer Type** (Ref. Hematologic)				
Solid	2.39 [-4.69 to 8.49]	0.57		

Abbreviations: IC, Informed Consent; SDOH, Social Determinants of Health; CI, Confidence Interval.
[a]Comprehension of voluntariness domain score (range 0-100).
[b]Ethnicity, Satisfaction with informed consent, Cancer type
[c]Significant variables in multivariable model are from the parsimonious multivariable model after backward elimination.
[d]Coefficient estimate, 95% CI and *P-value*, from linear mixed effects model.
[e]Unmarried includes single, divorced, widowed, and separated.
[f]Socioeconomic Status score (range 8-66).
[g]Health literacy adequate is defined as score (range 4-6).
[i]Satisfaction with IC score (range 7-21).

© 2023 Aristizabal P et al. *JAMA Network Open.*
