# Supplementary material for: Social Determinants of Health and Informed Consent Comprehension for Pediatric Cancer Clinical Trials
Source: JAMA Netw Open. 2023 Dec 11;6(12):e2346858. doi: 10.1001/jamanetworkopen.2023.46858 (PMC10714248; doi:10.1001/jamanetworkopen.2023.46858)
Supplement: Supplement 2. — Data Sharing Statement [file jamanetwopen-e2346858-s002.pdf]

# Data Sharing Statement

Aristizabal. Social Determinants of Health and Informed Consent Comprehension for Pediatric Cancer Clinical Trials. *JAMA Netw Open*. Published December 11, 2023.
doi:10.1001/jamanetworkopen.2023.46858

**Data**
**Data available:** Yes
**Data types:** Deidentified participant data
**How to access data:** De-identified data will be available from the corresponding author if requested, as needed
**When available:** With publication

**Supporting Documents**
**Document types:** None

**Additional Information**
**Who can access the data:** De-identified data will be available from the corresponding author if requested, as needed
**Types of analyses:** De-identified data will be available from the corresponding author if requested, as needed
**Mechanisms of data availability:** De-identified data will be available from the corresponding author if requested, as needed
**Any additional restrictions:** NA
